# Supplementary material for: A SARS-CoV-2 Spike Ferritin Nanoparticle Vaccine Is Protective and Promotes a Strong Immunological Response in the Cynomolgus Macaque Coronavirus Disease 2019 (COVID-19) Model
Source: Vaccines (Basel). 2022 May 4;10(5):717. doi: 10.3390/vaccines10050717 (PMC9145473; doi:10.3390/vaccines10050717)
Supplement: Supplementary file 1 [file vaccines-10-00717-s001.zip › vaccines-1702423-supplementary.pdf]

Supplementary Materials for

A SARS-CoV-2 Spike Ferritin Nanoparticle Vaccine is Protective and Promotes a Strong Immunological Response in the Cynomolgus Macaque Coronavirus Disease 2019 (COVID-19) Model

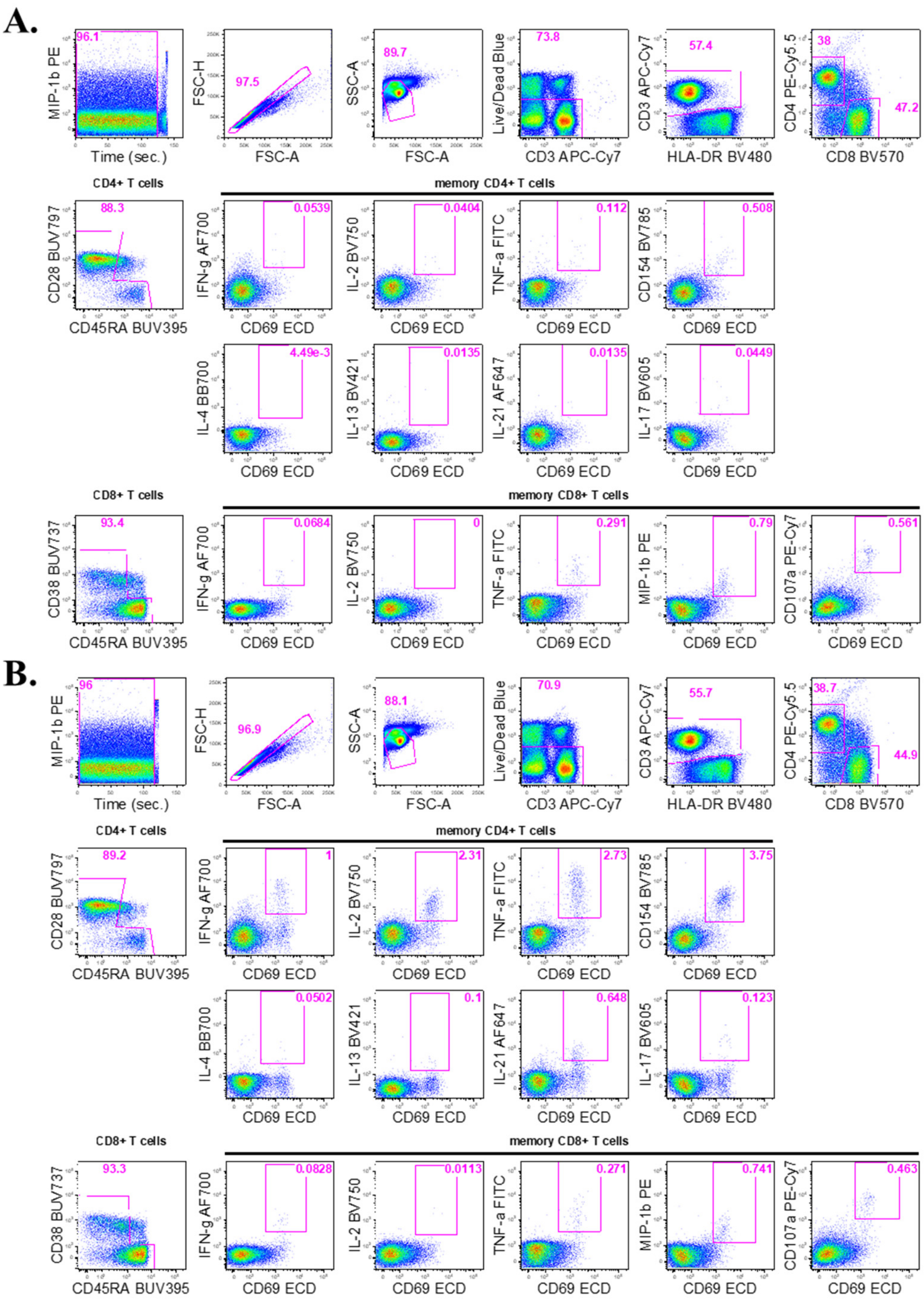

Figure S1. Representative FACS staining and gating strategy for T cell ICS. Flow cytometry gating applied to macaque PBMC T cell ICS data. FACS plots depict staining following stimulation with DMSO (**A**) or SARS-CoV-2 spike peptide pool #1 (**B**) for a representative animal at study week 6 following two immunizations with 50 µg SpFN adjuvanted with ALFQ. Sequential gates were applied from left to right (top row) to identify CD4+ and CD8+ T cells. Cytokine production was measured in total memory CD4+ (middle) and CD8+ (bottom) T cells by excluding the naïve CD28+ CD45RA+ population. Cytokine-positive cells were identified by co-expression of CD69.

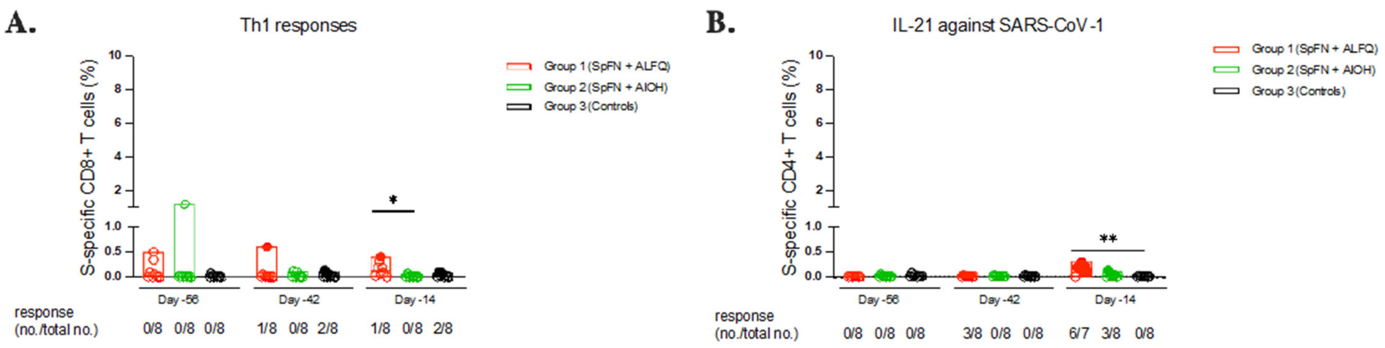

Figure S2. **SARS-CoV-2 and SARS-CoV-1 S-specific CD8 and CD4 T cell responses elicited by adjuvanted SpFN vaccination.** T cell responses were assessed by SARS-CoV-2 or SARS-CoV-1 spike peptide pool stimulation and intracellular cytokine staining of PBMC collected at study day -56 (pre-immune), study day -42 (2 weeks post-prime) and study day -14 (2 weeks post-boost). (**A**) SARS-CoV-2 S-specific memory CD8+ T cells expressing one or more Th1 cytokines (IFN-γ, TNF-α, and IL-2) is shown. () SARS-CoV-1 S-specific memory CD4+ T cells expressing IL-21 is shown. The fraction of animals within each group with a positive response following each vaccination is indicated. Significant differences between groups was assessed using a Kruskal-Wallis test followed by a Dunn's post-test (*$p < 0.05$, **$p < 0.01$).

Table S1. Origin and Physical Information

CM#	Group#	Vaccine	Species	Origin	Gender	Approximate Age (years) at Time of Challenge	Weight (kg) at Time of Challenge
2	1	SpFN + ALFQ	*Macaca fascicularis*	Asian	M	7.7	5.979
5	1	SpFN + ALFQ	*Macaca fascicularis*	Asian	F	5.6	2.935
7	1	SpFN + ALFQ	*Macaca fascicularis*	Asian	M	5.8	5.566
8	1	SpFN + ALFQ	*Macaca fascicularis*	Asian	F	5.7	4.836
9	1	SpFN + ALFQ	*Macaca fascicularis*	Asian	M	5.7	3.884
14	1	SpFN + ALFQ	*Macaca fascicularis*	Asian	F	6.0	3.449
21	1	SpFN + ALFQ	*Macaca fascicularis*	Asian	F	5.3	3.086
24	1	SpFN + ALFQ	*Macaca fascicularis*	Asian	M	7.6	11.91
1	2	SpFN + AlOH3	*Macaca fascicularis*	Asian	F	5.7	2.908
3	2	SpFN + AlOH3	*Macaca fascicularis*	Asian	F	7.0	3.352
4	2	SpFN + AlOH3	*Macaca fascicularis*	Asian	M	5.4	4.199
6	2	SpFN + AlOH3	*Macaca fascicularis*	Asian	F	8.4	3.297
10	2	SpFN + AlOH3	*Macaca fascicularis*	Asian	M	5.7	7.126
16	2	SpFN + AlOH3	*Macaca fascicularis*	Asian	M	5.4	7.731
18	2	SpFN + AlOH3	*Macaca fascicularis*	Asian	M	5.7	4.341
23	2	SpFN + AlOH3	*Macaca fascicularis*	Asian	F	5.5	2.899
11	3	PBS	*Macaca fascicularis*	Asian	F	5.6	3.115
12	3	PBS	*Macaca fascicularis*	Asian	M	8.2	9.099
13	3	PBS	*Macaca fascicularis*	Asian	F	5.7	2.864
15	3	PBS	*Macaca fascicularis*	Asian	F	6.3	3.426
17	3	PBS	*Macaca fascicularis*	Asian	M	5.7	4.303
19	3	PBS	*Macaca fascicularis*	Asian	F	5.1	3.439
20	3	PBS	*Macaca fascicularis*	Asian	M	9.5	8.778
22	3	PBS	*Macaca fascicularis*	Asian	M	5.6	5.667

Table S2. Summary of clinical disease findings

CM #	2	5	7	8	9	14	21	24	1	3	4	6	10	16	18	23	11	12	13	15	17	19	20	22
Group	1	1	1	1	1	1	1	1	2	2	2	2	2	2	2	2	3	3	3	3	3	3	3	3
Euthanasia Day	15	15	15	15	9	9	9	9	15	9	15	15	15	9	9	9	9	9	9	15	9	15	15	15
Hyperpyrexia[1]																			X		X		X	
Fever[2]					X	X												X	X	X	X	X	X	
Significantly Elevated Body Temp[3]	X				X	X		X		X							X	X	X	X	X	X	X	X
Diurnal Rhythm Disruption[4]	X		X			X				X		X												
Mild Hypoxia[5]						X		X				X											X	X
Tachycardia		X	X	X	X	X	X	X			X	X	X	X	X	X		X	X	X	X	X	X	X
Cough				X	X					X								X			X			
Increased Lung Opacity (# Affected Lobes)	X (1)		X (1)	X (1)^			X (1)^			X (4)	X (1)				X (2)				X (2)	X (2+)	X (4)			
Lung Infiltrates (# Affected Lobes)									X (1)	X (2)							X (1)			X (1)	X (4)	X (2)		
Abdominal Component to Breathing		X				X						X					X				X			
Piloerection								X	X	X		X		X	X		X	X		X	X			
Decreased Skin Turgor									X				X							X		X		X
Lymphadenopathy				X																X		X		
Stool Not Fully Formed/Liquid			X	X	X					X		X	X											
Anorexia[6]		X		X					X		X	X				X	X	X		X	X			
Reduced Consumption[7]				X	X				X	X	X	X		X		X	X	X		X	X			

*Mild hypoxia is defined as 90–94% SpO2

[1]Defined as a temperature greater than or equal to 3.0°C above baseline by telemetry, with baseline being the mean of data from Study Days -7 through -1 for a particular animal

[2]Defined as a temperature greater than or equal to 1.5°C above baseline for a duration of greater than 2 hours by telemetry

[3]Defined as a temperature greater than 3 standard deviations above baseline for a duration of greater than 2 hours by telemetry.

[4]Defined as a significant activity increase during the dark cycle, or a significant activity decrease during the light cycle, compared to baseline as measured by telemetry

[5]Defined as 90–94% SpO_2

[6]Defined as an absence of biscuit and enrichment consumption for one or more days OR an absence of biscuit consumption for 3 or more consecutive days

[7]Defined as either no evidence or notably reduced amounts of biscuit and/or fruit consumption for greater than or equal to 2 consecutive days

^Potential positional or rotational artifact

Table S3. Summary of Major Histopathologic Findings in the Respiratory Tract

Animal Number	Group	Alveolar inflammation	Perivascular inflammation	Peribronchiolar inflammation	Pleural inflammation and/or fibrosis	Nasal turbinate inflammation
CM 2	1	None	Mild to moderate; 2 of 8 sections affected	None	None	Minimal; 2 of 6 sections affected
CM 5	1	None	None	None	None	None
CM 7	1	None	None	None	None	None
CM 8	1	None	Minimal/1 of 8 sections affected	None	None	None
CM 9	1	Minimal; 1 of 8 sections affected	Minimal to mild; 6 of 8 sections affected	Mild; 2 of 8 sections affected	Mild; 2 of 8 sections affected	Minimal to mild; 2 of 6 sections affected
CM 14	1	Minimal/1 of 8 sections affected	Minimal to moderate; 3 of 8 sections affected	Mild/2 of 8 sections affected	None	None
CM 21	1	Minimal/2 of 8 sections affected	Minimal/3 of 8 sections affected	None	None	Minimal/1 of 6 sections affected
CM 24	1	None	None	None	None	Minimal; 1 of 6 sections affected
CM 1	2	None	Minimal/1 of 8 sections affected	None	Minimal/1 of 8 sections affected	Minimal - mild; 3 of 6 sections affected
CM 3	2	None	Minimal/2 of 8 sections affected	None	Minimal/1 of 8 sections affected	None
CM 4	2	Minimal; 1 of 8 sections affected	None	None	Mild; 1 of 8 sections affected	None
CM 6	2	Mild; 1 of 8 sections affected	Minimal - mild; 2 of 8 sections affected	None	Mild/1 of 8 sections affected	Mild - moderate; 3 of 6 sections affected

Table S3 (con't). Summary of Major Histopathologic Findings in the Respiratory Tract

Animal Number	Group	Alveolar inflammation	Perivascular inflammation	Peribronchiolar inflammation	Pleural inflammation and/or fibrosis	Nasal turbinate inflammation
CM 10	2	None	None	None	None	None
CM 16	2	Minimal to mild; 2 of 8 sections affected	Minimal to moderate; 4 of 8 sections affected	Mild to moderate; 2 of 8 sections affected	Mild; 2 of 8 sections affected	None
CM 18	2	None	Minimal to mild; 2 of 8 sections affected	Mild; 1 of 8 sections affected	None	None
CM 23	2	None	None	None	None	Minimal/1 of 6 sections affected
CM 11	3	Minimal - moderate; 7 of 8 sections affected	Mild - moderate; 2 of 8 sections affected	Mild - moderate; 3 of 8 sections affected	None	None
CM 12	3	Minimal to mild; 4 of 8 sections affected	Minimal to mild; 4 of 8 sections affected	None	None	None
CM 13	3	Moderate/1 of 8 sections affected	Minimal - moderate; 5 of 8 sections affected	Mild/2 of 8 sections affected	None	Minimal/1 of 6 sections affected
CM 15	3	Mild; 1 of 8 sections affected	Moderate; 1 of 8 sections affected	None	None`	Mild-moderate; 5 of 6 sections affected
CM 17	3	Mild; 2 of 8 sections affected; fibrin present	Mild; 2 of 8 sections affected	None	Mild/1 of 8 sections affected	None
CM 19	3	Minimal to mild; 2 of 8 sections affected	Minimal - mild; 4 of 8 sections affected	None	None	Minimal; 2 of 6 sections affected
CM 20	3	Mild; 1 of 8 sections affected	Mimial to mild; 4 of 8 sections affected	None	None	Minimal - 1 of 6 sections affected
CM 22	3	None	Minimal to mild; 2 of 8 sections affected	None	None	None

Table S4. Summary of histologic findings (excluding the lungs and nasal turbinates)

Animal Number	Group	Tracheobronchial Lymph Node	Axillary Lymph Node	Inguinal Lymph Node	Liver	GI Tract	Other
CM 2	1	Moderate lymphoid hyperplasia	Mild lymphoid hyperplasia	Medullary fibrosis	Hepatocyte atrophy, capsular fibrosis	Lymphoid hyperplasia of the ileocecolic lymph node	Testicular atrophy, urinary bladder and prostate gland infiltrate, interstitial nephritis
CM 5	1	Moderate lymphoid hyperplasia	Minimal lymphoid hyperplasia	Draining hemorrhage		Stomach inflammation	Urinary bladder infiltrate, ovarian cyst, skeletal muscle degeneration, necrosis and regeneration with inflammation
CM 7	1		Medullary fibrosis	Medullary fibrosis		Stomach inflammation	Splenic lymphoid hyperplasia, prostate gland infiltrate
CM 8	1	Mild lymphoid hyperplasia				Stomach inflammation	Adrenal gland mineralization
CM 9	1	Minimal lymphoid hyperplasia	Medullary fibrosis				Heart, trachea and prostate infiltrate
CM 14	1	Mild lymphoid hyperplasia					Interstitial nephritis, ovarian corpus amalacea
CM 21	1	Moderate lymphoid hyperpasia, medullary infiltrate		Medullary fibrosis		Stomach inflammation	Tracheal infiltrate, mesenteric lymphoid hyperplasia
CM 24	1		Granulomatous inflammation	Granulomatous inflammation		Cecal parasite	
CM 1	2	Marked lymphoid hyperplasia				Stomach inflammation, lymphoid hyperplasia ileocecolic LN	Splenic lymphoid hyperplasia, interstitial nephritis
CM 3	2	Mild lymphoid hyperplasia			Lymphocytic infiltrate		Splenic lymphoid hyperplasia
CM 4	2	Mild lymphoid hyperplasia	Medullary fibrosis		Lymphocytic infiltrate	Stomach inflammation, GALT lymphoid hyperplasia	Splenic lymphoid hyperplasia, tracheal infiltrate

Table S4 (con't). Summary of histologic findings (excluding the lungs and nasal turbinates)

Animal Number	Group	Tracheobronchial Lymph Node	Axillary Lymph Node	Inguinal Lymph Node	Liver	GI Tract	Other
CM 6	2		Mild lymphoid hyperplasia		Lymphocytic infiltrate	Stomach inflammation, lymphoid hyperplasia esophagus, duodenal infiltrate	Ovarian corpora amylacea
CM 10	2		Medullary fibrosis	Medullary fibrosis, draining hemorrhage		Peyer's patch lymphoid hyperplasia	Infiltrate in heart, urinary bladder and prostate gland; interstitial nephritis
CM 16	2		Reticuloendo-thelial cell hyperplasia	Medullary fibrosis, draining hemorrhage			Lymphoid hyperplasia in kidney, prostate gland infiltrate
CM 18	2	Lymphoid hyperplasia, medullary infiltrate	Lymphoid hyperplasia	Medullary fibrosis		Ileal and cecal parasite	Infiltrate in heart, prostate gland and sciatic nerve
CM 23	2					Stomach inflammation	Ovarian mineralization
CM 11	3		Minimal lymphoid hyperplasia				Skeletal muscle regeneration
CM 12	3			Draining hemorrhage		Stomach inflammation	Skeletal muscle degeneration & necrosis with inflamamtion
CM 13	3	Mild lymphoid hyperplasia					Thyroid gland infiltrate
CM 15	3	Mild lymphoid hyperplasia			Neutrophilic and eosinophilic infiltrate	Esophageal and cecal infiltrate, lymphoid hyperplasia ileocecolic LN	Heart degeneration with inflammation, urinary bladder and kidney infiltrate,
CM 17	3	Mild lymphoid hyperplasia			Mononuclear infiltrate	Stomach inflammation, cecal parasite	Prostate gland infiltrate
CM 19	3	Mild lymphoid hyperplasia					Interstitial nephritis
CM 20	3	Minimal lymphoid hyperplasia					Infiltrate in urinary bladder and corpus striatum
CM 22	3	Minimal lymphoid hyperplasia		Medullary fibrosis		Infiltrate in esophagus, cecal parasite	Heart infiltrate, interstitial nephritis

Table S5. Summary of clinical pathology findings – Vaccination Phase

CM #	2	5	7	8	9	14	21	24	1	3	4	6	10	16	18	23	11	12	13	15	17	19	20	22
Group	1	1	1	1	1	1	1	1	2	2	2	2	2	2	2	2	3	3	3	3	3	3	3	3
↑ WBC											X													
↑ NEUT											X			X										
↑ EOS											X													
↑ BAS											X													
↑ LYM		X						X											X					X
↑ MON		X																	X				X	
↑ ALT		X								X						X		X						
↓ ALB						X																		
↑ ALP											X													
↑ AST		X						X															X	
↑ AMY				X																				
↑ BUN		X															X							
↑ CRE							X																	
↑ TBIL														X										
↑ CK		X	X			X		X		X	X	X	X		X	X			X	X		X	X	
↑ CRP						X																		

X = % change from baseline (Study Day -56) of ≥30%

30–49% Δ

50–69% Δ

70–99% Δ

100–499% Δ

>500%

Table S6. Summary of clinical pathology findings – Challenge Phase

CM #	2	5	7	8	9	14	21	24	1	3	4	6	10	16	18	23	11	12	13	15	17	19	20	22
Group	1	1	1	1	1	1	1	1	2	2	2	2	2	2	2	2	3	3	3	3	3	3	3	3
↑ WBC					X		X		X	X	X	X		X				X		X		X	X	X
↑ NEUT	X				X		X	X	X	X	X	X		X	X				X	X		X	X	X
↑ EOS	X		X	X	X		X		X		X	X		X	X					X	X	X	X	X
↑ BAS					X				X		X	X		X						X		X	X	X
↑ LYM		X	X	X		X	X		X		X	X	X					X		X		X		X
↑ MON		X	X	X	X	X		X	X	X	X		X		X		X	X		X	X	X	X	X
↓ PLT			X	X							X					X								X
↑ ALT			X	X										X		X		X	X	X				
↑ ALP								X									X	X	X	X		X		X
↑ AST	X		X					X	X		X	X	X	X		X	X	X	X	X	X	X		
↑ GGT																								X
↑ GLU			X					X																
↑/↓ AMY								X									X						X	
↑ BUN	X			X																				
↑ CRE		X	X			X														X				
↑ TBIL								X			X		X				X			X			X	
↑ CK	X	X	X	X	X	X	X	X	X	X	X	X	X	X	X	X		X	X	X	X	X	X	
↑ CRP		X	X	X	X	X		X				X					X	X	X	X	X	X	X	

X = % change from baseline (average of Study Days -4 and 1 per animal) of ≥30%

30-49% Δ

50-69% Δ

70-99% Δ

100-499% Δ

500-999% Δ

>1000% Δ
